# Investigating *CD11c* expression as a potential genomic biomarker of response to TNF inhibitor biologics in whole blood rheumatoid arthritis samples

**DOI:** 10.1186/s13075-015-0868-y

**Published:** 2015-12-14

**Authors:** Samantha Louise Smith, Stephen Eyre, Annie Yarwood, Kimme Hyrich, Ann W. Morgan, A. G. Wilson, John Isaacs, Biologics in Rheumatoid Arthritis Genetics and Genomics Study Syndicate 6, Darren Plant, Anne Barton

**Affiliations:** Arthritis Research UK, Centre for Genetics and Genomics, Centre for Musculoskeletal Research, Manchester Academic Health Sciences Centre, the University of Manchester, Manchester, UK; Arthritis Research UK, Centre for Epidemiology, Centre for Musculoskeletal Research, Manchester Academic Health Sciences Centre, The University of Manchester, Manchester, UK; Leeds Institute of Rheumatic and Musculoskeletal Medicine, University of Leeds and NIHR Leeds Musculoskeletal Biomedical Research Unit, Leeds Teaching Hospitals NHS Trust, Leeds, UK; UCD School of Medicine and Medical Science, Conway Institute, University College Dublin, Dublin, Ireland; NIHR Newcastle Biomedical Research Centre, Newcastle upon Tyne NHS Foundation Trust and Newcastle University, Newcastle upon Tyne, UK; Biologics in Rheumatoid Arthritis Genetics and Genomics Study Syndicate, Centre for Genetics and Genomics Arthritis Research UK, Stopford Building, Manchester, UK; NIHR Manchester Musculoskeletal BRU, Central Manchester Foundation Trust and University of Manchester, Manchester Academic Health Science Centre, Manchester, UK

**Keywords:** Transcriptomics, Treatment response, Biomarker, Rheumatoid arthritis, Pharmacogenomics

## Abstract

**Introduction:**

Gene expression profiling is rapidly becoming a useful and informative tool in a much needed area of research. Identifying patients as to whether they will respond or not to a given treatment before prescription is not only essential to optimise treatment outcome but also to lessen the economic burden that such drugs can have on healthcare resources. In rheumatoid arthritis (RA), there is of yet no genetic/genomic biomarker which can accurately predict response to TNF inhibitor biologics prior to treatment, despite much interest in this area. Multiple studies have reported findings on potential candidate genes; however, due to relatively small sample sizes or lack of sufficient validation, results have been disappointingly inconsistent. The aim of this research was to further explore the predictive value of a previously reported association between *CD11c* expression and response to the TNF inhibitor biologics, adalimumab and etanercept.

**Methods:**

Real-time qPCR was performed using whole blood RNA samples obtained from seventy-five rheumatoid arthritis patients about to commence treatment with a TNF inhibitor biologic drug, whose response status was determined at 3-month follow-up using the EULAR classification criteria. Relative quantification of *CD11c* using the comparative C_T_ method outputted differential expression between good-responders and non-responders as a fold-change.

**Results:**

Relative expression of *CD11c* in patients receiving TNF inhibitor biologics yielded a decrease of 1.025 fold in good-responders as compared to non-responders (p-value = 0.36). Upon stratification of patients dependent upon the specific drug administered, adalimumab or etanercept, similar findings to the full cohort were observed, decreases of 1.015 (p-value = 0.33) and 1.032 fold (p-value = 0.13) in good-responders compared to non-responders, respectively.

**Conclusion:**

The results from this study reveal that *CD11c* expression does not correlate with response to TNF inhibitor biologics when tested for within pre-treatment whole blood samples of rheumatoid arthritis patients.

## Introduction

Rheumatoid arthritis (RA) is a systemic autoimmune disorder of unknown aetiology affecting around 400,000 adults in the UK [1] and is characterised by chronic inflammation, joint swelling and fatigue. Early diagnosis and aggressive treatment are known to correlate with improved long-term outcome, ultimately resulting in a better quality of life [2–5]. Within the last decade, the treatment strategy for RA has progressed considerably, particularly since the introduction of biologic therapies, which greatly benefit patients who do not respond to traditional disease-modifying anti-rheumatic drugs (DMARDs) [6]. Although effective, biologic agents are costly (approximately £10,000 per patient per year) and are reserved in many countries for those patients with moderate to severe RA, who have previously failed to respond to more than one DMARD, including methotrexate (MTX) [1]. However, up to 40 % of RA patients receiving biologic agents fail to respond satisfactorily.

Clinical characteristics such as age, gender and concurrent DMARD use only contribute 15–17 % of the variance observed in treatment response [7]. Hence, identifying predictive biomarkers to help clinicians select an effective drug early in the disease course has been highlighted as a research priority. In the field of oncology, transcriptional profiling is routinely utilized to stratify treatment regimens; for example, over-expression of the *HER2/neu* gene in breast cancer patients is used to inform the prescription of trastuzumab (Herceptin), whilst oestrogen receptor expression is associated with a better response to tamoxifen [8–10]. It is possible, therefore, that expression biomarkers of response could be identified in other fields of medicine. Indeed, expression of *CD11c* (*ITGAX*), an integrin molecule involved in cell adhesion and chemotaxis [11] has shown evidence for association with response to treatment with a TNF inhibitor drug (TNFi) in RA patients. It was reported that increased expression of *CD11c* prior to treatment significantly correlates with good response to adalimumab monotherapy (*p* value <0.0001); with sensitivity of 100 % and specificity of 91.7 % [12].

However, the previously observed association was conducted using RA monocytes and due to the impracticality of using such a sample source in clinical settings, it is likely to hamper the adoption of this biomarker for routine clinical use. Ideally, for a predictive biomarker to be useful in a clinical setting, it should be possible to test the biomarker in a biological sample that is readily available, acquired through a minimally invasive procedure (resulting in the least possible distress to the patient) and requiring minimal processing following collection. Hence, whole blood would be an ideal source for a biomarker. We therefore set out with the aim not to replicate the previously reported observation, but to (1) establish whether expression of *CD11c* is detectable in whole blood samples collected from RA patients about to commence treatment with a TNFi biologic drug and (2) correlate pre-treatment whole blood expression levels with response to TNFi biologic drugs both as a whole and sub-grouped by the drug received (both adalimumab and etanercept were tested).

## Methods

### Patients

Seventy-five patients with RA were selected for analysis from the Biologics in Rheumatoid Arthritis Genetics and Genomics Syndicate (BRAGGSS), which recruits patients from over 50 contributing centres across the UK, who are about to commence treatment with biologic drugs for the first time, as described in detail previously [7]. Inclusion criteria were as follows: participants were Caucasian, over the age of 18 years, fulfilled the 1987 American College of Rheumatology (ACR) criteria for RA and gave written informed consent. Upon recruitment, patients were asked to provide blood samples for the collection of laboratory and serological data and provide psychological and clinical information. As part of the study, patients are prospectively followed for 12 months and provide further samples/information at months 3, 6 and 12. As such, the 28-joint count disease activity score (DAS28) using four variables (the number of tender and swollen joints, erythrocyte sedimentation rate (ESR)/C-reactive protein (CRP) and patient global assessment score) could be recorded prior to and at 3, 6 and 12 months [13]. The BRAGGSS study was approved by NRES Committee North West - Greater Manchester South (REC Ref: 04/Q1403/37).

For this study, patients were selected if they were about to commence treatment with the TNFi biologic drugs, adalimumab or etanercept, and if they showed either a good response or no response to therapy at 3-month follow up. Clinical efficacy of the TNFi biologic drug was assessed at 3-month follow-up using the established European League Against Rheumatism (EULAR) classification criteria. Good-responder patients were defined by having a DAS28 ≤ 3.2 but also having decreased from the pre-treatment DAS28 by >1.2. A non-responder was defined as having a DAS28 score that did not decrease by >0.6 or was between 0.6–1.2 but also with an end score >5.1. Moderate responders were excluded from this study.

### Blood collection

Pre-treatment whole blood samples were collected into either Tempus ™ Blood RNA Tubes (3 ml) (Applied Biosystems, Foster City, CA, USA) or PAXgene Blood RNA collection tubes (2.5 ml) (Qiagen, Valencia, CA, USA). Following collection, samples were shipped to the central processing laboratory at the Arthritis Research UK Centre for Genetics and Genomics (ARUK-CoGG) where they were logged onto the laboratory information management system (LIMS) and stored in an allocated freezer location at –80 °C until RNA extraction.

### RNA extraction

RNA extraction kits were used to extract total RNA from whole blood samples following the manufacturer’s instructions: i.e., Tempus™ Spin RNA Isolation Kit (Applied Biosystems, Foster City, CA, USA) and PAXgene RNA Isolation Kit (Qiagen, Valencia, CA, USA). In both cases an on-column DNase digestion step was performed to remove any potential genomic DNA contamination. Quantification was performed using the Nanodrop ND-1000 (Thermo Scientific, Waltham, MA, USA) and assessment of RNA integrity was assessed using the 2100 Bioanalyzer (Agilent, Santa Clara, CA, USA). Total RNA with 260/280 and 260/230 ratios in the range of 1.8–2.2 and RNA integrity number (RIN) >5 were taken forward for analysis.

### cDNA Synthesis and RT-qPCR

Total RNA was reverse transcribed into high-quality single stranded cDNA using the High Capacity cDNA Reverse Transcription Kit (Applied Biosystems, Foster City, CA, USA) following the manufacturer’s instructions. Briefly, 10 μl total RNA was reverse transcribed in a 10-μl master-mix solution containing MultiScribe™ Reverse Transcriptase and placed in a thermal cycler programmed according to the manufacturer’s instructions.

Quantitative real-time PCR (RT-qPCR) was performed using the QuantStudio™ 12 K Flex real-time qPCR system (Applied Biosystems, Foster City, CA, USA) which employs TaqMan based technology. Relative expression levels of *CD11c* were determined by normalising *CD11c* mRNA transcripts against *GAPDH* and *ACTB* mRNA transcripts (housekeeping genes (HKGs)). All TaqMan® gene expression assays used were assays-by-demand supplied by Applied Biosystems: *ACTB* Hs99999903_m1, *GAPDH* Hs99999905_m1 and *ITGAX* (*CD11c*) Hs00174217_m1. All experiments were performed in triplicate, under PCR amplification conditions in accordance with the manufacturer’s instructions.

### Statistical analysis

All statistical analysis was performed using the data analysis and statistical software programme STATA/SE 11.2 (StataCorp LP, College Station, TX, USA) [14]. The comparative cycle threshold (C_T_) method was used to relatively quantify *CD11c* expression against an average of the HKGs resulting in *CD11c* expression being presented as a fold-change in good responders as compared to non-responders [15]. Subsequently, normalised *CD11c* expression values were used in a logistic regression model; both univariate and multivariate models were tested (the multivariate model including baseline characteristics with a *p* value <0.05 for comparison between the response phenotypes as covariates). The analysis was performed in two stages. First, the analysis was performed on the full cohort and second, the analysis was repeated following stratification of patients into sub-groups based on the TNFi biologic drugs they received.

## Results

Seventy-five patients receiving TNFi biological therapy were included in this study. The summarised cohort characteristics of these patients can be seen in Table 1 and are typical of patients with active and severe disease. Similar baseline characteristics were observed for good responders and non-responders except that good responders were slightly younger (*p* value = 0.023) and presented with a lower health assessment questionnaire (HAQ) score (*p* value = 0.026). Using the EULAR classification criteria, 41 patients were classified as TNFi responders and 34 patients as TNFi non-responders at 3 months following commencement of treatment (Table 1).

**Table 1:** Summarised cohort/stratified baseline characteristics of patients receiving biologic drugs, analysed for *CD11c* expression by qPCR

	Full cohort (n = 75)			Etanercept (n = 50)			Adalimumab (n = 25)		
Cohort characteristics	NR (n = 34)	R (n = 41)	*P* value	NR (n = 25)	R (n = 25)	*P* value	NR (n = 9)	R (n = 16)	*P* value
Gender, female, n (%)	27 (79.4 %)	31 (75.6 %)	0.695^a^	20 (80 %)	20 (80 %)	1.00^a^	7 (77.8 %)	11 (68.8 %)	0.629^a^
Age, baseline, years, mean (SD)	59.3 (12.3)	53.3 (10.3)	0.023^b^	61.2 (12.2)	54.86 (11.4)	0.065^b^	54.3 (11.7)	50.7 (8.2)	0.258^b^
Concurrent DMARDs, n (%)	28 (82.4 %)	36 (90 %)	0.338^a^	22 (88 %)	21 (84 %)	0.684^a^	6 (66.7 %)	15 (100 %)	0.017^a^
Baseline DAS28, median (IQR)	6.2 (5.4–6.6)	5.8 (5.5–6.3)	0.131^c^	6.2 (5.8–6.6)	5.9 (5.5–6.2)	0.156^c^	6.6 (5.6–7.03)	5.6 (5.3–6)	0.101^c^
3 month DAS28, median (IQR)	5.65 (5.4–6.2)	2.15 (1.6–2.5)	<0.001^c^	5.6 (5.3–5.9)	2.3 (1.9–3)	<0.001^c^	6.6 (5.3–6.8)	1.95 (1.6–2.2)	<0.001^c^
Baseline TJC, median (IQR)	16 (10–19)	13 (10–16)	0.205^c^	14 (10–18)	13 (10–15)	0.515^c^	18 (16–24)	13 (10–19)	0.157^c^
Baseline SJC, median (IQR)	8.5 (4–13)	9 (6–13)	0.701^c^	7 (4–11)	9 (6–13)	0.268^c^	11 (9–15)	8.5 (6–13)	0.211^c^
Baseline ESR, median (IQR), n	33 (14–44), 26	18 (14–33), 35	0.11^c^	32 (23–41), 21	18 (10–34), 23	0.158^c^	34 (10–60), 5	20.5 (14–31), 12	0.712^c^
Baseline HAQ, median (IQR), n	2.06 (1.38–2.3), 22	1.5 (1.13–2), 32	0.026^c^	2.1 (1.9–2.3), 17	1.4 (1.1–1.6), 19	0.006^c^	1.9 (1.3–2.1), 5	1.8 (1.1–2.4), 13	0.96^c^
Tempus™ Blood Tube, n (%)	21 (61.8 %)	22 (53.4 %)	0.48^a^	15 (60 %)	14 (56 %)	0.774^a^	6 (66.67 %)	8 (50 %)	0.420^a^

^a^Calculated using chi-square test. ^b^Calculated using two independent samples *t* test. ^c^Calculated using Wilcoxon-Mann–Whitney test. Samples were grouped according to treatment response. *NR* non-responders, *R* responders, *DAS28* disease activity score in 28 joints, *SJC* swollen joint count, *TJC* tender joint count, *ESR* erythrocyte sedimentation rate, *HAQ* health assessment questionnaire

**Fig. 1:**
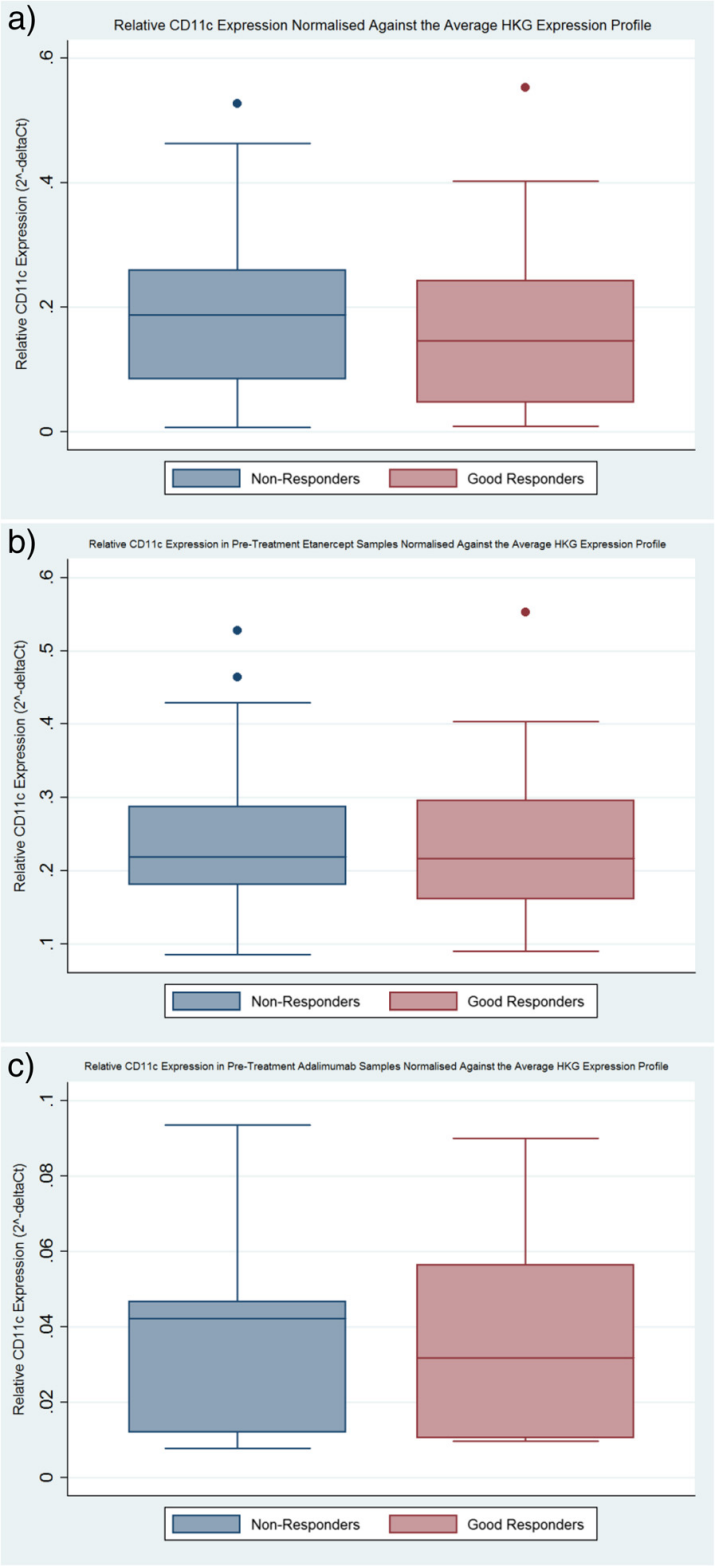
Relative *CD11c* expression stratified by response phenotype. Pre-treatment *CD11c* expression normalised to the average housekeeping gene (*HKG*) profile and grouped according to treatment response (presented as 2^-ΔC^ ^t^). Measured in 75 total RNA samples from patients about to receive anti-TNF treatment (**a**), in 50 total RNA samples from patients about to receive the anti-TNF etanercept (**b**) and in 25 total RNA samples from patients about to receive the anti-TNF adalimumab (**c**)

Good-responder and non-responder patients receiving TNFi biologic drugs demonstrated similar baseline *CD11c* expression profiles when normalised against the average HKGs profile using the comparative C_T_ method (see Fig. 1), equating to a 1.025-fold decrease in good responders as compared to non-responders (multivariate *p* value = 0.36; using age at baseline and baseline HAQ score as covariates).

As the previously reported association was reported in patients receiving adalimumab, the analysis was repeated after stratifying patients based on the type of treatment received; this equated to 25 adalimumab patients (16 good responders and 9 non-responders) and 50 etanercept patients (25 good responders and 25 non-responders). Stratified cohort characteristics are listed in Table 1. No significant associations were observed in the stratified analysis. Adalimumab patients yielded a 1.015-fold decrease in good-responders as compared to non-responders (multivariate *p* value = 0.33, using concurrent DMARD use as a covariate) and etanercept patients yielded a 1.032-fold decrease in good-responders as compared to non-responders (multivariate *p* value = 0.13, using baseline HAQ score as a covariate).

The previous studies initial exploratory analysis of seven RA patients treated with adalimumab showed that higher *CD11c* expression correlated with good response. However further analysis revealed that the association was only observed in patients receiving monotherapy and was not observed in adalimumab-treated RA patients when adalimumab was prescribed with MTX, as is common in clinical practice. Further analysis restricted to patients on monotherapy was undertaken in the current study where possible; however, this did not lead to any significant findings at the 5 % significance threshold, though the small group numbers (n = 7) included in the analysis limited the conclusions that could be drawn.

## Discussion

This study has employed a candidate gene approach to further explore the potential predictive capability of expression of a previously reported treatment response gene, *CD11c*, using a clinically applicable sample source (as opposed to the original sample source of monocytes). To achieve this, expression of *CD11c* was measured in whole blood samples collected at baseline from RA patients about to undergo treatment with TNFi biologic drugs. However, no evidence for association was detected for TNFi drug therapy as a class or after sub-grouping by specific TNFi agents.

A strength of this study was the large sample size tested relative to previous treatment response gene expression profiling studies and the use of samples from extreme ends of the response spectrum, which resulted in greater power to detect the same effect size as previously reported in the study of adalimumab-treated patients [12]. For example, in the original study, after initial identification of *CD11c* expression associating with treatment response in 7 patients by microarray analysis, validation was conducted in 27 patients from an independent cohort by RT-PCR [12]. By contrast, the current study of 75 RA samples is, to the best of our knowledge, one of the largest and most well-powered treatment response studies of gene expression conducted to date in RA.

A major difference between this study and the original study was the testing of *CD11c* expression within whole blood rather than in monocytes; therefore, as previously stated, the previous association has not been faithfully tested. The discovery of the correlation between *CD11c* expression and adalimumab response may be wholly cell-type-specific and due to the heterogeneous nature of whole blood any signal could be confounded by saturation from other cells. However, a recent study [16] demonstrated that CD11c expression is quantifiable in whole blood and we therefore elected to test whole blood, as this sample type would be most readily translated to clinical benefit, whereas isolation of specific cell populations is currently impractical in a clinical setting. Further experiments in an enriched monocyte cell population and whole blood samples will be needed to explore this further.

Another reason for the lack of association could be due to the use of the EULAR classification criteria to define response to treatment within this study, whereas in the original study patients were classified according to the ACR response criteria. Both outcome measures are well-validated and therefore commonly used to assess response to treatment; however, there is debate as to the concordance between them, with the use of which measure being at the discretion of the researcher. For example, one study reported that the concordance between classifying an ACR20 and EULAR response was high (with all 94 ACR20 patients found in the EULAR response group), however, in contrast, concordance between the ACR50 and EULAR response groups was poor (with only 34 ACR50 patients (out of 53) found within the EULAR group) [17]. Of note, ACR scores could not be calculated within the current study.

Another consideration to take into account is that different TaqMan® primers were used within this study, compared to the original. However, both primer sets detected the same transcript so this is not the reason for the lack of replication. Furthermore, it could be that the association between *CD11c* expression and response is only applicable to patients receiving adalimumab monotherapy, as the signal was lost upon administration of concomitant MTX. However, according to the National Institute for Health and Care Excellence (NICE) guidelines for the treatment of patients with RA in the UK, biologic drugs should be given in conjunction with MTX and only in patients who have proven intolerant or developed an adverse reaction should a biologic be given as monotherapy [18]. This would therefore limit the usefulness of *CD11c* as a treatment response biomarker, as those receiving adalimumab monotherapy will only represent a small minority of the overall number of patients receiving biologic drugs.

Given the success of gene expression profiling in guiding therapy decisions in the field of oncology, expression profiling is currently being explored as a biomarker predictive of treatment response to other therapies, including biologic treatments used for RA [19–23]. However, results so far have been disappointing as inconsistencies in the reported findings have been observed. This is due to a number of reasons, including differences in study design (type of tissue used, treatment/dose received, classification criteria applied or the platform used to generate the results) or lack of statistical power. Future well-powered studies of treatment response are therefore needed in RA and whole blood remains an attractive tissue to test for biomarkers in the clinical setting. Considering this, it would be ideal to agree upon a standardised method of generating data in treatment response studies before a clinical biomarker can be identified and sufficiently validated for use in the clinic.

## Conclusion

We hypothesised that the expression profile of *CD11c*, previously reported to be associated with response to adalimumab therapy when tested in monocytes, would also correlate with response to TNFi agents with enough discriminatory power to be detectable in whole blood samples. However, our study shows that expression of *CD11c* in baseline whole blood samples does not correlate with treatment outcome in etanercept-treated or adalimumab-treated RA patients.

## Abbreviations

ACR: American College of Rheumatology
ACTB: beta-actin
BRAGGSS: Biologics in Rheumatoid Arthritis Genetics and Genomics Study Syndicate
C_T_: cycle threshold
DAS28: disease activity score in 28 joints
DMARD: disease modifying anti-rheumatic drug
ESR: erythrocyte sedimentation rate
EULAR: the European League Against Rheumatism
GAPDH: glyceraldehyde 3-phosphate dehydrogenase
gDNA: genomic DNA
HAQ: health assessment questionnaire
HKG: housekeeping gene
ITGAX: integrin alpha X (complement component 3 receptor 4 subunit)
MTX: methotrexate
NICE: National Institute for Health Care Excellence
qPCR: quantitative polymerase chain reaction
RA: rheumatoid arthritis
RIN: RNA integrity number
SJC: swollen joint count
TJC: tender joint count
TNF: tumour necrosis factor
TNFi: tumour necrosis factor inhibitor
